# Editorial: The Role of Mutations, Stresses and Post-Translational Modifications in the Structure and Function of Small Heat Shock Proteins and Their Relationship with Different Human Diseases

**DOI:** 10.3389/fmolb.2022.922959

**Published:** 2022-06-08

**Authors:** Alok Kumar Panda, Ashis Biswas

**Affiliations:** ^1^ School of Applied Sciences, KIIT Deemed to be University, Bhubaneswar, India; ^2^ School of Basic Sciences, Indian Institute of Technology Bhubaneswar, Bhubaneswar, India

**Keywords:** small heat shock proteins, chaperone function, post-translational modification (PTM), mutations, redox stress, human diseases, holdase

Small heat shock proteins (sHSPs) are the proteins whose monomeric molecular weight ranges between 12 and 42 kDa (Narberhaus 2002; Basha, O'Neill et al., 2012). They are ubiquitously expressed (Haslbeck and Vierling 2015). Most of them are β-sheet proteins and possess a large dynamic oligomeric assembly (Haslbeck and Vierling 2015). The presence of a highly conserved “α-crystallin domain” (central domain) is the common structural feature of most of the members of the small heat shock protein family (Kriehuber, Rattei et al., 2010). It is noteworthy to mention here that some sHSPs (HSP15, HSP31, and HSP33) do not contain “α-crystallin domain.” These sHSPs contain only N-terminal and C-terminal domains. HSP15 is a ribosome-associated sHSP and possesses a different functional role than all other sHSPs. On the other hand, HSP31 and HSP33 are redox-regulated molecular chaperones (Narberhaus 2002). In fact, “α-crystallin domain” containing sHSPs (sometimes termed as “α-HSPs”) also exhibit holdase/chaperone function. Some well known α-HSPs [Archeal sHSPs such as HSP16.5 from *Methanococcus jannaschii* and HSP26 from *Saccharomyces cerevisiae*, α-crystallin and HSP27 (mammalian sHSPs), plant sHSP (HSP16.9 from wheat) and mycobacterial sHSP (HSP16.3 from *Mycobacterium tuberculosis*)] whose chaperone function has been investigated extensively. α-HSPs generally interact with partially folded target proteins and prevent their aggregation under stressed conditions (Bakthisaran, Tangirala et al., 2015). Many sHSPs are biomarkers and are considered as important drug target for combating diseases like cancer and *tuberculosis*. The chaperone function of these sHSPs often play pivotal role in regulating different human diseases. For example, the chaperone function of some mammalian sHSPs (αA-crystallin, αB-crystallin and HSP27) plays important role in controlling the diseases like cataract, cancer and cardiovascular disease. Whereas, the molecular chaperone function of some mycobacterial sHSPs (*M. leprae* HSP18 and *M. tuberculosis* HSP16.3) is detrimental for mankind (Jee, Katoch et al., 2008). Because their chaperone function actually helps the corresponding pathogen to survive inside infected hosts. In fact, a plethora of studies successfully affirm the intrinsic involvement of sHSPs in several human diseases.

The objective of this Frontiers Research Topic was to bring together a cross-section of reports on the effect of mutations, stresses and post-translational modifications on the structure and function of different sHSPs and how these structure-function relationships relate with different human diseases. Since mutations in many sHSPs are associated with different human diseases like cataract, cancer, leprosy and cardiovascular diseases, the effect of such mutations on the structure and function of sHSPs and their relationship with diseases is an important and fundamental issue to be addressed. Similarly, many post-translational modifications (such as glycation, phosphorylation, deamidation and truncation) and different stresses (especially UV and redox stress) also have a large impact on the structure and chaperone function of many sHSPs. But how such structural and functional modulations in various sHSPs are influencing various important diseases, are some intriguing issues to be addressed. Understanding these conundrums is important for identifying new targets and developing new set of therapeutics to treat/control deadliest diseases such as heart disease, cancer, lung disease and cerebrovascular disease.

For this Research Topic, we received many submissions from which 7 articles by 43 authors across the globe were published. The articles included not only the original research papers but also contained reviews and mini-reviews.

Gao et al. reviewed the role of endoplasmic recticulum stress (ER stress) on the gut enterocyte and their relationship with intestinal diseases. This review explained about the unfolded protein response that the cell activates in the face of ER stress and the consequent mechanisms and proteins involved in stabilizing the ER homeostasis under stressed conditions. The authors also illustrated the role of ER in various cellular functions and the changes brought about in these functions during the ER stress. Furthermore, they discussed about a variety of proteins, including small heat shock proteins that are involved in the ER stress and the therapeutic potential of these proteins to inhibit gastrointestinal diseases.

The mini-review by Chakraborty et al. vividly elucidated the structural and functional aspects of HtrA2, which decipher the underlying mechanisms responsible for its bi-functional chaperone-protease activity. Being a serine protease, HtrA2 is involved in multiple cellular networks and pathological activities. Despite homologous oligomeric structure and overall structural fold, minor conformational alterations and dynamic enzymatic regulation *via* an allosteric mode of action contribute to functional diversity of this protein. Its N-terminal tetrapeptide (AVPS) motif binds and cleaves inhibitor of apoptosis proteins (IAPs), causing cell death and providing a therapeutic target. Both the N- and C-terminal activation of many substrates by HtrA2 has been linked to diseases like neurological disorders and cancer. Apart from its potential therapeutic roles, HtrA2’s unique structural features, such as multimodal activation, intermolecular PDZ-protease crosstalk, and an allosterically modulated trimeric active-site ensemble further make it an interesting protease to study extensively.

The mini-review by Roy et al. summarized the existing knowledge on archaeal small heat shock proteins (sHSPs) in conjunction with the information on bacterial and eukaryotic homologs. The review represented the details related to the various facets of sHSPs in maintaining proteostasis, the dynamics and heterogeneity in oligomerization, and the crosstalk between sHSPs with other cellular chaperones. The authors have presented a case that archaeal sHSPs may constitute a useful model to understand the structure-function relationship of eukaryotic homologs that are recalcitrant to such studies.

The review by Tedesco et al. summarized the structural and biochemical effects of disease related mutations in human small heat shock proteins and their functional consequences. It is clear from this review that mutations in human sHSPs affect those tissues in which sHSPs themselves are predominantly or exclusively expressed, giving rise to a clear clinical phenotype in affected patients. However, mutations in human sHSPs with a pleiotropic activity may be related to different or multiple phenotypes. Importantly, mutations include missense, nonsense, and frameshift mutations that may possibly affect human sHSPs protein expression and stability, post-translational modifications, dynamics in oligomerization and interaction with client proteins, thus altering the activity of human small heat shock proteins. At the end of the review, various therapeutic strategies have been discussed to counteract the onset or progression of human sHSPs-related diseases.

The mini-review submitted by Nandi et al. mainly focused on various ATP binding sHSPs like αA-crystallin, αB-crystallin, HSP27, HSP20, *Mycobacterium leprae* HSP18 and *Mycobacterium tuberculosis* HSP16.3. The authors explained about the impact of ATP on the structure and function of these human and mycobacterial sHSPs in a comprehensive manner. How ATP-sHSPs interaction can influence the onset of several human diseases is also discussed in this mini-review. The authors have presented that the improved chaperone function of many sHSPs in the presence of ATP eventually helps in controlling various important diseases. They also highlighted how ATP induced enhanced chaperone function of certain sHSPs affected adversely some other human diseases (like tuberculosis and leprosy). Possible development of ATP based therapeutics is also discussed nicely in this mini-review.

The review contributed by Rajeswaren et al. summarized the recent findings on sHSPs (especially HSPB4 and HSPB5) in different retinal diseases (glaucoma, diabetic retinopathy, and age-related macular degeneration). The authors provided a general description of the structure and major cellular functions of sHSPs in this review. Their role in specific retinal diseases, highlighting their regulation, role in pathogenesis, and possible use as therapeutics, is discussed. This important review univocally depicted that the chaperone function and anti-apoptotic properties of sHSPs appear to be important in preventing neurodegeneration and vascular abnormalities in the retina.

Lastly, the work done by Santhoshkumar and Sharma revealed that methylglyoxal modification reduces the aggregation propensity of G98R mutant human αA-crystallin. In this study, the authors initially showed that αA-G98R forms less compact and high molecular weight complexes with the client proteins as compared to wild-type human αA-crystallin, and the resulting complexes continue to increase in size over time. Interestingly, when αA-G98R modified with low concentrations of methylglyoxal, the chaperone activity of this mutant protein have been improved due to the stabilization of chaperone (αA-G98R)-substrate complex. The authors at the end have suggested that the uncontrolled long-term interaction amongst mutant subunits and client proteins may be the reason behind the formation of presenile cataract, and the initial stabilization of αA-G98R by substrate proteins may delay the appearance of this congential cataract.

Altogether, we hope that this collection of reviews and original research for this Research Topic provides new perspectives and insights into the wide range of connections between “structural and functional modulations of sHSPs due to mutations, post-translational modifications and stresses” with different important human diseases.
